# The Landscape of Malaria in a Five-Decade Institution in the State of Amazonas: Historical Trends, Scientific Contributions, and Research Priorities

**DOI:** 10.1590/0037-8682-0563-2025

**Published:** 2026-07-17

**Authors:** Anne Cristine Gomes de Almeida, Camila Fabbri, Marcelo Augusto Mota Brito, José Diego Brito-Sousa, Fernando Almeida-Val, Joabi Rocha do Nascimento, Flor Ernestina Martinez-Espinosa, Patrike Machado Barbosa, Elder Augusto Guimaraes Figueira, Myrna Barata Machado, Vanderson de Souza Sampaio, Stefanie Costa Pinto Lopes, Wuelton Marcelo Monteiro, Marcus Vinicius Guimaraes de Lacerda, Gisely Cardoso de Melo

**Affiliations:** 1 Fundação de Medicina Tropical Doutor Heitor Vieira Dourado, Manaus, AM, Brasil.; 2 Universidade do Estado do Amazonas, Manaus, AM, Brasil.; 3 Universidade Federal do Amazonas, Manaus, AM, Brasil.; 4 Fundação Oswaldo Cruz Amazônia, Instituto Leônidas & Maria Deane, Manaus, AM, Brasil.; 5 Secretaria Municipal de Saúde, Manaus, AM, Brasil.; 6 Fundação De Vigilância Em Saúde Do Amazonas - Drª Rosemary Costa Pinto, Manaus, AM, Brasil.; 7 Instituto Todos pela Saúde, São Paulo, SP, Brasil.; 8 Duke University, Duke Global Health Institute, Durham, USA.

**Keywords:** Malaria, Plasmodium vivax, Plasmodium falciparum, Elimination plan, Clinical research, Biomedical research

## Abstract

Malaria continues to be a major public health issue in the Brazilian Amazon, with the state of Amazonas accounting for approximately 40% of all national cases. The region presents diverse epidemiological scenarios and different infrastructure conditions, which hinder the implementation of diagnostic, treatment, management, and control strategies. Through dedicated control, surveillance and research initiatives, considerable progress has been made in reducing the incidence of cases, hospitalizations and deaths attributed to the two endemic protozoan species, *Plasmodium falciparum* and *Plasmodium vivax*. This article provides an overview of the key research and surveillance endeavors undertaken in the state of Amazonas. These efforts aim to achieve the objectives outlined in the national malaria elimination plan, enhance the understanding of the disease’s pathophysiology and associated comorbidities, and leverage the advances and contributions of research groups within the state, which are focused on comprehending vector behavior and developing effective control strategies, creating more efficient diagnostic tools and researching and developing safe and effective drugs for treatment, relapse prevention and the elimination of transmission reservoirs.

## INTRODUCTION

Malaria continues to be a major public health challenge in the state of Amazonas, a region that has historically been responsible for most of the cases reported in Brazil^1^ ^,^ ^2^. In addition to control efforts, scientific studies conducted in Amazonas have provided critical insights into the pathophysiology of *Plasmodium vivax*, evaluated new therapeutic agents through clinical trials, and advanced the understanding of resistance mechanisms and host-parasite interactions using genomic, transcriptomic and metabolomic approaches^3^ ^-^ ^8^. This manuscript reviews the historical trends, scientific contributions and future research priorities that define the evolving landscape of malaria in the state of Amazonas.

## TEMPORAL EVOLUTION AND CURRENT SITUATION OF MALARIA IN AMAZONAS AND BRAZIL

In Brazil, malaria transmission is largely confined to its Amazon region, which accounts for about 99.9% of national cases^2^ ^,^ ^9^. In 2025, 120,549 cases of malaria were recorded, with 117,775 in the Amazon region and 80% of the total reported cases caused by *P. vivax*. In the state of Amazonas, 61,752 malaria cases were registered in 2025, with the majority of cases reported in the municipalities of São Gabriel da Cachoeira, Manaus and Barcelos^2^ ^,^ ^10^. 

**FIGURE 1:**
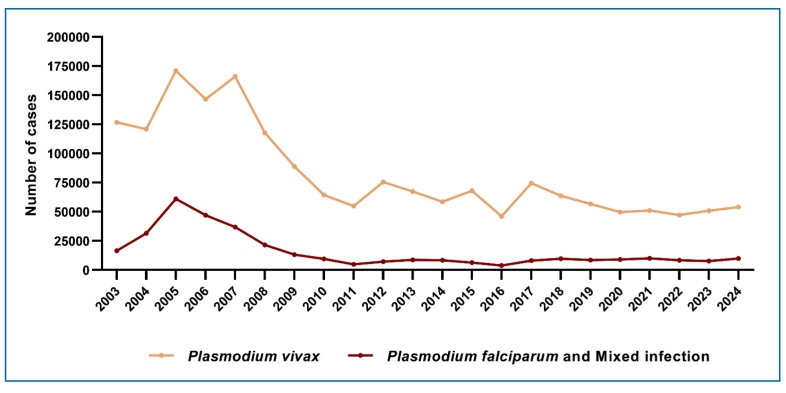
Twenty-year trend of reported *Plasmodium vivax* and *P. falciparum* cases in Amazonas state. Caption: Annual number of reported malaria cases caused by *Plasmodium vivax* (yellow line) and by *P. falciparum* plus mixed infections (red line). The figure illustrates the marked decline in malaria incidence over the last two decades, particularly after the mid-2000s, with *P. vivax* remaining the predominant species throughout the period. Data source: SIVEP-Malária, Brazilian Ministry of Health. Created in GraphPad Prism version 9.0.

According to data from the *Sistema de Informação de Vigilância Epidemiológica* (SIVEP-Malaria; Malaria Epidemiological Surveillance System), in 2003, 232,065 malaria cases were registered in the state of Amazonas, 171,019 caused by *P. vivax* and 61,046 by *Plasmodium falciparum* or mixed malaria, the highest frequency recorded in the historical series (Figure 1)^2^. Direct engagement between *Fundação de Vigilância em Saúde - Dr. Rosemary Costa Pinto* (FVS-RCP) and municipal health services led to a 16.5% reduction in malaria cases in 2006^11^ ^,^ ^12^. 

In 2007, the Multiyear Malaria Control Action Plan (2007-2010) achieved a 70.5% reduction in cases, reaching levels considerably lower than in the previous decade. Later, the Plan for the Intensification of Malaria Control for *P. falciparum*, implemented in 2016, further strengthened control actions, leading to the lowest number of cases in the state’s historical record-49,929 autochthonous infections, a 32.9% decrease compared with the previous year^11^. Key improvements include the expansion of the diagnostic network, acquisition of microscopes and rapid tests, strengthening of river and land logistics, and qualification of human resources^13^. The incorporation of more effective therapeutic regimens, such as tafenoquine (TQ) for *P. vivax* and artesunate plus mefloquine for *P. falciparum*, contributed to greater adherence and a reduction in the disease. The implementation of the MalariaTratat® app supported therapeutic standardization and clinical safety. In vector control, the distribution of Long-Lasting Insecticide-Protected Nets (LLINs) was crucial for reducing vector density and transmission^14^. The combination of these structured interventions was fundamental to changing the epidemiological profile in the state^15^ ^-^ ^17^.

In 2022, Brazil launched the National Malaria Elimination Plan (PNEM), outlining strategic guidelines to eliminate the disease by 2035. At the federal level, the coordinating unit is responsible for ensuring universal access to timely diagnosis and treatment, promoting epidemiological surveillance as a core intervention, and supporting states in strengthening entomological surveillance and vector control activities^11^. Aligned with the national strategy, the Malaria Elimination Plan 2023-2035, developed by FVS-RCP, adopts a phased implementation model to foster coordination among municipalities and promote decentralized, shared governance^12^. 

## MAPPING OF AVAILABLE RESOURCES FOR DISEASE CONTROL

The Malaria Elimination Program in Amazonas is executed in close collaboration with nine health regions and 62 municipalities and is adapted to local accessibility and cultural contexts. Decentralization of health services in Amazonas enables municipalities to tailor malaria control strategies to local contexts, improving response effectiveness. However, structural inequalities (such as disparities in infrastructure, workforce, and social conditions) limit performance^12^.

**FIGURE 2:**
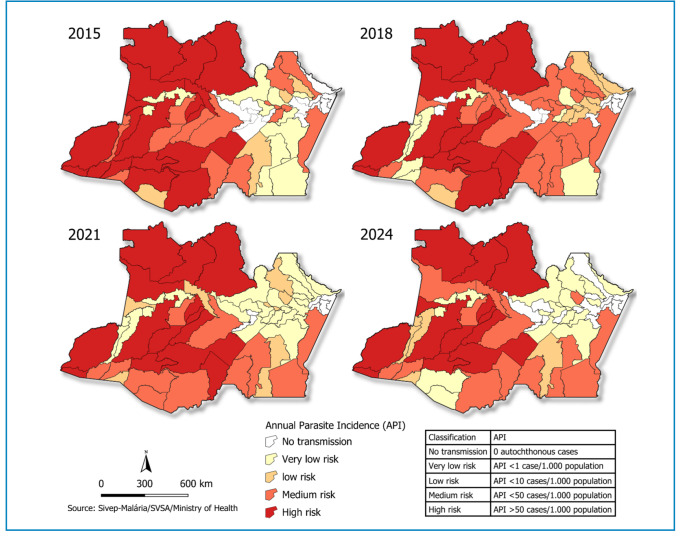
Spatial distribution of the annual parasite incidence among the municipalities of the state of Amazonas. Caption: The maps illustrate the temporal evolution of malaria transmission intensity, highlighting the progressive reduction and spatial reorganization of high-risk areas over time, alongside the persistence of transmission hotspots in specific regions of the state. High incidence rates are consistently maintained in the northern region of the state, especially in the area known as Alto Rio Negro and Baixo Amazonas and Triângulo regions, as well as in the municipality of Atalaia do Norte, located in the Alto Solimões region. Data source: SIVEP-Malária, Brazilian Ministry of Health. Created in ArcGIS.

**FIGURE 3:**
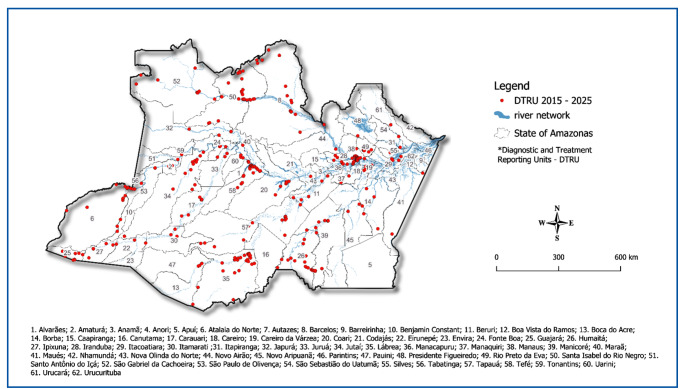
Network of malaria diagnosis and treatment reporting units in the state of Amazonas. Caption: Red dots indicate the location of active Diagnostic and Treatment Reporting Units (DTRU), illustrating the geographic coverage of malaria diagnosis and treatment services across the state. The river network is shown in blue, highlighting the strong association between DTRUs distribution and fluvial accessibility. Municipal boundaries and the state outline are displayed in grey. Numbers correspond to municipalities, listed in the figure caption. It is worth noting that the historical records include units registered from 1966 through 2025, highlighting the consolidation of the malaria surveillance infrastructure in Amazonas over nearly six decades. Created in ArcGIS.

High incidence rates are consistently maintained in the northern region of the state, especially in the area known as Alto Rio Negro, which is historically recognized as the region with the highest concentration of the Indigenous population in the state (Figure 2)^2^. The spatial distribution and temporal evolution of the network of Diagnosis and Treatment Reporting Units (DTRUs) in Amazonas are provided by an information system from SIVEP-Malaria. Between 2015 and 2025, 1,690 active DTRUs were registered across the state (Figure 3)^2^ ^,^ ^12^. 

In Amazonas, malaria in indigenous territories is influenced by socio-environmental determinants and logistical barriers that hinder access to diagnosis and adherence to treatment, fundamental aspects for interrupting disease transmission^11^ ^,^ ^12^. In border areas with Colombia and Peru, continuous mobility and low integration of surveillance increase transmission^18^. Added to this is illegal mining, which alters the environment, favoring vector breeding sites and the displacement of workers, generating persistent foci in indigenous and riverside lands^12^ ^,^ ^19^.

### Vector control

Malaria transmission in Brazil is sustained mainly by *Anopheles darlingi*, which is the dominant vector in the Amazon basin. In addition, secondary vectors such as species within the *An. albitarsis* complex, *An. nuneztovari*, also contribute to local transmission in specific ecological contexts, although with lower efficiency and more heterogeneous patterns of infectivity^14^. 

In Amazonas, vector control strategies have advanced with the introduction of LLINs that maintain residual activity for up to six months, especially in hard-to-reach areas, such as Indigenous, riverine and rural communities to strengthen the actions of the PNEM^11^ ^,^ ^12^. Furthermore, control activities include Indoor Residual Spraying (IRS), thermal fogging and the installation of insecticide-treated screens, implemented according to the stratification of locations based on probable sites of infection and SIVEP-Malaria data^12^. Such environmental changes (extreme flooding and drought events) have the potential to expand vector distribution, prolong transmission seasonality and complicate entomological control, representing a significant threat to the sustainability of malaria elimination strategies^20^.

## EVOLUTION OF SCIENTIFIC KNOWLEDGE REGARDING DISEASE CONTROL AND TREATMENT

### *Anopheles* spp*.* Colonies: a pillar for malaria research and control

The first *Anopheles* spp. colony established at *Fundação de Medicina Tropical Dr. Heitor Vieira Dourado* (FMT-HVD) was of *An. aquasalis*, which was selected for its adaptability to laboratory conditions. Martins-Campos et al. (2018)^21^ showed that this species can be reliably infected with blood from both symptomatic and asymptomatic *P. vivax* patients.

In addition, the genomic sequencing of *An. aquasalis* revealed a genome enriched in genes related to ion transport and osmoregulation-adaptations to brackish environments-as well as immune and detoxification genes^22^. Furthermore, with a transcriptomic profile, Santana et al. (2019)^23^ demonstrated that *P. vivax* induces an autophagic response involving genes such as beclin, autophagy related (*Beclin*) and autophagy-related protein 8 (*Apg8*).

Studies by Fabbri et al. (2021, 2023)^6^ ^,^ ^24^ confirmed *An. aquasalis* as a valuable model for *P. vivax* transmission-blocking assays. The first study identified Malaria Box compounds with promising gametocytocidal activity. Later, methylene blue proved even more effective, completely blocking transmission in membrane feeding assays after gametocyte exposure. The *An. darlingi* colony was established in 2021 using specimens from a hyperendemic malaria region in Amazonas, Brazil. Initial studies with this colony focused on vaccine development^25^ ^,^ ^26^ and the assessment of the impact of three combination therapies on *P. vivax* transmission in both *An. darlingi* and *An. aquasalis* ^27^.

### Clinical contributions to the understanding of the pathophysiology of *P. vivax* malaria

Clinical and experimental studies in the Brazilian Amazon reveal that *P. vivax* infection can cause severe disease even with low parasitemia, but still uncommon compared to *P. falciparum* ^28^ ^,^ ^29^. Amazon findings are similar to other endemic places, such as India, Thailand-Myanmar border and even Republic of Korea^30^ ^-^ ^32^. Also, the vivax malaria infection can lead to the involvement of multiple organ systems, affecting the liver, lungs and spleen^33^ ^-^ ^35^ (Table 1). Many authors have discussed the pathophysiology of malaria, highlighting the increase in various cytokines that trigger an immune-inflammatory response and correlate with disturbances in hemostasis. Table 1 also summarizes these findings^36^ ^-^ ^40^.

**TABLE 1:** Clinical Severity, Relapse Patterns and Biomarker Profiles of *Plasmodium vivax* in the Brazilian Amazon.

Study	Marker/Disorder	Interpretation/Association	System or organ involved
Alexandre et al. (2010)^28^	Severe anemia, acute renal failure	The study emphasizes that parasitemia cannot be considered a severity factor, as even patients with low parasite density presented clinical manifestations consistent with severe malaria. Among the main findings, severe anemia was the most relevant, in addition to ARDS and renal failure. Jaundice was also observed; however, the WHO currently does not consider isolated jaundice a criterion for severe malaria.	Hematologic / Hemostasis
Mendonça et al. (2015)^33^	Hepcidin, IL-6, IL-10, IFN-γ, IL-1β, IL-12p70, TNF	Hepcidin was significantly higher in patients with hyperbilirubinemia and severe *P. vivax* malaria compared to mild cases. Positive correlations were found between hepcidin and IL-6, IL-10, and IFN-γ, and between hepcidin and parasitemia in mild cases. Network analysis identified IL-1β, IL-6, IL-12p70, TNF and IFN-γ as major inflammatory interaction nodes.	Liver / Systemic inflammation
Elizalde-Torrent et al. (2018)^35^	Splenic rupture	Splenic rupture during acute *P. vivax* infection is rare but likely underdiagnosed, emphasizing the need for early imaging and careful monitoring during treatment, especially in endemic areas with limited diagnostic resources.	Spleen
Val et al. (2017)^34^	Respiratory distress	Among 587 hospitalized *P. vivax* patients, 30 (5.1%) developed respiratory complications. Of these, 13 (43.3%) had severe manifestations. Intensive care was required for 12 (40%) patients, and 5 (16.6%) died. Most respiratory complications occurred after antimalarial administration (60%) and progressed rapidly.	Lungs
Costa et al. (2020)^37^	Platelet count, MPV, PDW, IL-1β, IL-2, IL-12p70	71% of *P. vivax* patients presented thrombocytopenia, which was not associated with parasitemia levels. IL-2 and IL-12p70 were reduced, while IL-1β was elevated, and these cytokine changes correlated with platelet indices.	Hematologic / Hemostasis
Dos-Santos et al. (2020)^36^	Platelet count, sCD40L, TPO, IL-11, E-selectin, ICAM-1, vWF	Among *P. vivax* patients from Manaus, 87% presented thrombocytopenia with a mild increase in mean platelet volume. Plasma levels of sCD40L, IL-11, TPO, E-selectin, ICAM-1 and vWF were significantly elevated. Network analysis revealed strong positive correlations between platelet and endothelial activation markers.	Hematologic / Endothelial system
Oliveira dos Santos et al. (2020)^38^	TRP, KYN, KYN/TRP ratio, IFN-γ, IDO1.	Serum KYN and KYN/TRP ratios were significantly increased during acute infection, indicating activation of the IFN-γ-IDO1 pathway. Patients with primary infections showed higher KYN and IFN-γ levels compared to those with previous malaria episodes.	Immune regulation
Chaves et al. (2016)^39^	IL-10, IL-6, TNF, CD4⁺ and CD8⁺ T lymphocytes, activation marker CD69⁺	Recurrent infections were characterized by a decrease in CD4⁺ and CD8⁺ T-cell counts and lower expression of activation marker CD69⁺ compared to primary infections. Plasma IL-10 was elevated in recurrent cases and positively correlated with both CD4⁺/CD8⁺ ratio and number of previous malaria episodes.	Immune regulation
Shuan Laco et al. (2025)^40^	IFN-γ in memory T cells (CD3^+^CD4^+^CD45RO^+^and CD3^+^CD8^+^CD45RO^+^T cells)	PBMCs were stimulated ex vivo with the conserved PvMSP1_19_ antigen and intracellular production of IFN-γ was quantified in memory T cells. CD3^+^CD4^+^CD45RO^+^T cells exhibited sustained IFN-γ response against PvMSP1_19_, demonstrated lower parasite density and improved normalization of hemoglobin levels and recovery of platelet counts.	Immune regulation

**Caption:** Summary of the main biomarkers and pathophysiological associations identified in studies conducted at the Fundação de Medicina Tropical Dr. Heitor Vieira Dourado (FMT-HVD) on *P. vivax* malaria in the Brazilian Amazon. The table highlights key associations between inflammatory, immune regulation, hematologic and endothelial alterations and their corresponding organ systems or physiological pathways. **ARDS:** Acute Respiratory Distress Syndrome; **CD:** cluster of differentiation; **CD45RO:** leukocyte common antigen; **IL:** interleukin; **ICAM:** intercellular adhesion molecule; **IDO1:** indoleamine 2,3-dioxygenase 1; **IFN:** interferon; **KYN:** kynurenine; **MPV:** mean platelet volume; **PDW:** platelet distribution width; **PBMC:** peripheral blood mononuclear cells; **PvMSP1:** *P. vivax* merozoite surface protein 1; **sCD40L:** soluble CD40 ligand; **TNF:** tumor necrosis factor; **TPO:** thrombopoietin; **TRP:** tryptophan; **vWF:** von Willebrand factor; **WHO:** World Health Organization.

In addition to the influence of host and environmental factors on infection dynamics, nutritional status also plays a role in malaria outcomes. Benzecry et al. (2016)^41^ found no link between micronutrient deficiencies and the incidence of *P. vivax* malaria; however, Ferreira et al. (2015)^42^ confirmed that while nutritional status does not affect infection risk it does influence prognosis, and Monteiro et al. (2016)^43^ highlighted that recurrent infections may worsen nutritional status through inflammation and anorexia. Furthermore, *P. vivax* infection impairs cognition in different age groups. Tapajós et al. (2019)^44^ observed reduced cognitive scores in children, while Pessoa et al. (2022)^45^ reported lasting impairments in executive function in the elderly, indicating that *P. vivax* affects both nutritional and neurological health in endemic populations.

### Malaria in pregnancy in the Amazon region

Malaria in pregnant women in the Amazon region constitutes a significant public health problem. The prevalence of malaria in pregnant women in Manaus, Amazonas, is less than 1% when using microscopy, but 8.4% when tested using molecular methods^46^. *P. vivax* predominates among pregnant women (> 80%), although the proportion of *P. falciparum* infections is higher among pregnant women than in women of childbearing age. Adolescents and primigravidae present risks such as the risk of miscarriage and preterm birth^47^.

In pregnant women from endemic areas, antibodies against *P. vivax* variant interspersed repeat (VIR) protein and duffy binding protein (PvDBP) correlate with exposure and protection^48^ ^,^ ^49^. Studies also revealed distinct T-cell responses and elevated pro- and anti-inflammatory cytokines, in which C-C motif chemokine ligand 11 (CCL11) was linked to higher hemoglobin and Interleukin-4 Protein (IL-4) to lower birth weight, highlighting complex immune regulation during infection^50^ ^,^ ^51^.

The most frequent effect of malaria on pregnant women is the intensification of maternal anemia^47^. Moreover, about 57% of pregnant women who experience a malaria episode have recurrent parasitemia during pregnancy, increasing the risk of miscarriage and preterm birth. Placental ultrasonographic assessments showed an increase in placental thickness^52^, especially in *P. vivax* malaria^53^. The lack of radical cure during pregnancy, given the contraindication of primaquine in this period, increases maternal and neonatal morbidity and healthcare costs, underscoring the need for effective public policies to prevent and manage malaria during pregnancy^47^ ^,^ ^54^.

### Molecular basis of physiopathology: studies based on -omics approaches

Molecular profiling approaches, including metabolomics and transcriptomics, have advanced the understanding of *Plasmodium* spp. biology and host interactions^55^ ^,^ ^56^. Studies conducted with patients at FMT-HVD with vivax malaria showed distinct metabolic profiles in infections caused by chloroquine (CQ) - resistant *P. vivax*, with glycerophosphocholine metabolism being significantly lower in the CQ-resistant group compared to patients with CQ-sensitive parasites^57^. Recurrent *P. vivax* infections were associated with metabolic disturbances involving eicosanoids, vitamin B6, tyrosine, alkaloid biosynthesis, and alterations in butanoate, aspartate, asparagine, and N-glycan metabolism^8^. In addition, CQ-based therapies impact amino acid and redox metabolism, and dihydroartemisinin-piperaquine-based therapies affect lipid and mitochondrial pathways^58^. These results indicate distinct metabolomic signatures that are influenced by clinical status and treatment, which may guide future strategies for malaria control and therapy.

Regarding transcriptomic studies at FMT-HVD, researchers have described morphological and molecular alterations in the medullary tissue of individuals with *P. vivax* malaria. Their evidence points to dyserythropoiesis and inefficient erythropoiesis during acute malaria. The authors also contributed to the elucidation of the molecular bases of anemia and the behavior of *Plasmodium* spp. in this tissue, defining the bone marrow as an important niche for the maturation and maintenance of disease transmission^7^ ^,^ ^59^.

## BARRIERS AND FACILITATORS FOR DISEASE CONTROL AND TREATMENT

### G6PD screening in the Amazon: from bench to the bedside

Glucose-6-phosphate dehydrogenase (G6PD) deficiency, predominantly the African variant, affects ~5% of the Brazilian Amazon population^60^ ^,^ ^61^. The enzymatic deficit compromises antioxidant defenses, causing acute hemolysis after 8-aminoquinoline use in malaria patients. Hemolytic anemia linked to primaquine (PQ) use is well documented, with severe outcomes and economic burden^62^ ^,^ ^63^. Without routine screening, diagnosis typically occurs after hemolytic events, as testing is limited to reference labs and point-of-care implementation remains difficult.

In the Amazon, quantitative Standard™ G6PD outperforms the qualitative CareStart™ test, especially for intermediate results and TQ eligibility (>70% activity)^64^ ^,^ ^65^. Obstacles to implementation include remote locations, test supply, training and cultural perceptions^63^ ^,^ ^64^. G6PD-deficient patients must still receive an 8-week weekly PQ regimen despite TQ use^65^ ^,^ ^66^.

### Updates on uncomplicated vivax malaria treatment and prevention of relapses

Relapses and recurrences in vivax malaria result from dormant liver stages that may reactivate after the primary infection and are mostly influenced by transmission intensity and adherence to radical cure with 8-aminoquinolines^65^ ^,^ ^67^. Therefore, the data reported in the literature are associated with therapeutic regimens based on the use of PQ and, more recently, TQ^65^ ^,^ ^67^ ^,^ ^68^. A study from the Brazilian Legal Amazon identified 17% potential *P. vivax* recurrences, with a median time to recurrence of 69 days^68^. A review from 2023 found in a pooled analysis across 18 endemic countries, recurrence by day 90 ranged from 5.8% to 14.0%, depending on primaquine supervision, with no evidence that a single country disproportionately drove the recurrence signal^67^.

Brazil has led efforts in the prevention of *P. vivax* relapses. TQ, a single-dose analog of PQ with a longer half-life, showed superior efficacy to daily PQ when combined with CQ, with similar safety^4^ ^,^ ^5^. Poor adherence to daily PQ supports its replacement in endemic regions^69^.

In 2022, TQ was assessed in Manaus and Porto Velho, with prior G6PD testing. This study showed that G6PD testing promoted appropriate treatment or non-treatment for*P. vivax*radical cure with TQ in 99.7% of cases and with PQ in 88.7% of cases^16^. Qualitative surveys showed high acceptance of the new recommendations among healthcare professionals and users^70^ ^,^ ^71^. The use of TQ proved more effective than PQ in preventing relapses up to 180 days after infection and delaying the time until the first relapse^72^.

The Brazilian Ministry of Health, following the approval by the Brazilian National Committee for the Incorporation of Technologies, implemented single dose TQ with prior G6PD testing for uncomplicated *P. vivax* malaria, prioritizing indigenous areas and regions where gold mining occurs, as well as strategic Amazonian municipalities within the PNEM^17^.

### Innovations in malaria diagnostics

Studies conducted at FMT-HVD have investigated new methods for diagnosing malaria. Almeida et al. (2020)^73^ found a high proportion of asymptomatic individuals with subpatent parasitemia, after implementation of the polymerase chain reaction (qPCR) technique with the target 18S ribosomal RNA gene (*18S rRNA*), as well as gametocytemia that was detectable only by quantitative real-time reverse transcription polymerase chain reaction (qRT-PCR), using the target genes *P. vivax* and *P. falciparum* ookinete surface protein (*Pvs25* and *Pfs25,* respectively). Via qPCR with ultrasensitive target mitochondrial cytochrome oxidase 1 gene (*Pv-mtCOX1*), Barbosa et al. (2020)^74^ observed early subpatent recurrence of *P. vivax*. A study using a microscopy system that employs machine-learning-based image analysis (EasyScan Go), on Giemsa-stained blood films, demonstrated good results in relation to the performance levels required by the World Health Organization^75^ ^,^ ^76^. Another study evaluated a test for the detection of hemozoin, which uses the Gazelle™ device, with a magnetic-optical principle, which had good sensitivity and specificity^77^. These results emphasize the importance of innovative methodologies for diagnosing malaria in endemic areas.

### Improving the access to specialized health: TELEMAL+

Aiming to optimize the access of malaria patients to specialized consultations, the TELEMAL+ telehealth platform has been established. Its primary function is to connect the population and health professionals from other municipalities and remote regions to infectious disease specialists allocated at the FMT-HVD and FVS-RCP in Manaus^78^.

Additionally, the platform provides a collection of training material covering the diagnosis, treatment and management of malaria, HIV, arboviruses and other febrile diseases. This material undergoes a process of continuous updating and expansion, aligned with the epidemiological and health demands of the Amazonian population^78^.

### Influence of pharmacogenetics on therapeutic response

In recent years, the variability of genes encoding antimalarial metabolizing enzymes has been widely studied, main encoders of cytochrome P450 enzymes^79^. The pharmacogenetics related to hypnozoiticidal drugs is of particular importance due to the endemicity of *P. vivax*. Studies conducted in Amazonian populations^80^ ^-^ ^89^ have shown that variants of the cytochrome P450 2D6 (*CYP2D6*) and enzyme monoamine oxidase A (MAO-A) gene are associated with increased risk of recurrences or decrease in time to recurrence after PQ treatment. Phenotypes of ultrarapid metabolization of CYP2D6 enzyme were more frequent in patients who did not have treatment failure after using PQ and individuals deficient in the G6PD enzyme with rapid or ultrarapid phenotype of CYP2D6 and cytochrome P450 2C19 (CYP2C19) enzymes had higher levels of hemolysis markers^86^ ^,^ ^87^. 

A study showed cytochrome P450 2C8 (*CYP2C8*) wild-type individuals achieved greater reduction of gametocytes than low-activity allele carriers^80^. Also, it was reported that individuals with polymorphisms in *CYP2C8* often had recurrence in less than 42 days post-treatment, suggesting recrudescence due to CQ failure^81^. On the other hand, another study did not find an association between the presence of *CYP2C8,* cytochrome P450 3A4 (*CYP3A4*) and cytochrome P450 3A5 (*CYP3A5*) genotypes with early recurrences of *P. vivax* ^84^. Regarding TQ, no relationship has been demonstrated between genotypes associated with slow metabolization and recurrences by *P. vivax* ^90^ ^,^ ^91^. 

### Antimalarial resistance in the Brazilian Amazon

Current evidence indicates the presence of CQ-resistant *P. vivax* in the Brazilian Amazon^92^ ^,^ ^93^; however, confirmed resistance rates remain low and vary over time and location^93^. Importantly, CQ resistance in *P. vivax* is not unique to the Amazonas region and has been documented in several endemic areas worldwide, particularly in Southeast Asia and Oceania^9^ ^,^ ^94^. Studies in the Brazilian Amazon observed that alterations in the number of copies of the *P. vivax* CQ resistance transporter (*pvcrt*) gene were found in CQ-resistant isolates *in vivo* ^95^. Increased levels of expression of the *pvcrt* and *P. vivax*multidrug resistance (*pvmdr1*) genes were also observed in CQ-resistant isolates *in vivo* ^3^. The resistance rate of *P. vivax* to CQ in the Brazilian Amazon fluctuates, ranging from 5 to 11.5%^93^ ^,^ ^96^. Regarding *ex vivo* CQ resistance, a recent study with a pharmacogenetic and experimental approach found sensitive isolates when tested against CQ and desethylchloroquine^84^. Investigations carried out in the Brazilian Amazon have so far not found mutation in the *P. falciparum* Kelch 13 (*pfk13*) gene associated with partial resistance to artemisinin-based combination therapies (ACTs)^97^ ^,^ ^98^.

### Control of transfusion-transmitted malaria

Transfusion-Transmitted Malaria continues to be a challenge in endemic areas^99^, where cases of asymptomatic infection and submicroscopic parasitemia are common^100^ ^,^ ^101^. Brazilian blood bank regulations in endemic areas defer donors who have had malaria in the past 12 months, recent fever, or recent travel to high-risk areas^102^.

Currently, in Brazil, the presence of *Plasmodium* spp. infection is evaluated via laboratory screening using a new generation of the NAT PLUS HIV/HBV/HCV/Malaria (NAT-PLUS) kit, developed by Bio-Manguinhos/FIOCRUZ and implemented in the blood banks in 2022, after a study in Amazonas state^102^ ^,^ ^103^. The kit uses the detection of the target gene *18S rRNA* genus-specific of the parasite, in minipools of deoxyribonucleic acid (DNA) samples from six donors, obtained after processing the donor’s plasma^104^. After this implementation, studies have already reported cases of donors considered suitable for clinical screening, but who presented *Plasmodium* spp. infection detected by the NAT PLUS kit, with the infections coming from endemic locations and from the Brazilian extra-Amazon region^105^ ^-^ ^107^, emphasizing the importance of molecular screening in hemovigilance. 

## PRIORITY RESEARCH AGENDA FOR THE NEXT 10 YEARS IN THE CONTEXT OF THE AMAZON

Following the trend of other Latin American regions, Amazonas has advanced through malaria coordinated elimination policies. Scientific efforts, including *Anopheles* spp. Colonies, clinical, molecular, and -omics studies have expanded the understanding of the pathophysiology, diagnosis and control of *P. vivax*. The introduction of G6PD testing, tafenoquine, and molecular screening in blood banks has strengthened radical cure and hemovigilance. Additionally, to sustain the reduction in malaria cases and severity, it is crucial to continue widespread use of rapid tests in remote areas and ongoing efforts to improve prevention, diagnosis, and treatment among vulnerable populations such as indigenous groups, riverine communities, and mining workers, building on the progress made over the past decade.

Achieving malaria elimination requires tackling insecticide resistance, strengthening vector control and mosquito net use, advancing drugs and diagnostics, responsibly managing natural resources, and involving health professionals, stakeholders, and the public. Success also depends on addressing inequalities, climate change, new biological threats, and aligning scientific advances with public health priorities.

Financial, logistical, and research investments have been made to achieve the elimination goals, reducing the disease’s impact on the local population. Considering the PNEM^11^, the Amazonas team’s research agenda for the next years is described in Table 2.

**TABLE 2:** Malaria research priorities in Amazonas for the next 10 years.

**1. Partnership with the Brazilian public health system**
Inclusion of malaria in the list of diseases covered by the federal program “Healthier Brazil”, strengthening access to rapid diagnosis and timely treatment;
Expanding access to radical treatment for *Plasmodium vivax* with tafenoquine and primaquine, with prior testing for G6PD deficiency to ensure safe use;
Adequate implementation of pediatric formulations of tafenoquine and ACTs to ensure treatment efficacy in children.
**2. Treatment optimization and new tools for elimination**
Investigation of the efficacy and usability of ivermectin as a transmission blocking tool (BOHEMIA Protocol);
Study of tafenoquine dose adjustment based on patient weight for efficacy optimization (TADORE Protocol);
Investigation of the efficacy of the combination of tafenoquine with artesunate mefloquine for universal treatment of malaria (EFFORT Protocol);
Screening of new compounds for malaria treatment (Malaria Box);
Efficacy and safety of different treatment regimens using primaquine for radical cure of *P. vivax*;
Search for new markers for monitoring antimalarial resistance;
Assessing the appropriate time to replace chloroquine with ACTs as a first-line treatment for radical cure;
**3. Innovation in diagnostics**
Development and implementation of ultrasensitive rapid tests for the detection of *Plasmodium falciparum* and *P. vivax*, including asymptomatic infections (malRDT Protocol);
Searching for new candidate serological markers for the development of new diagnostic tools;
Implementation and expansion of the use of ultrasensitive molecular markers for the detection of submicroscopic malaria in areas of low endemicity and in the investigation of transfusion-transmitted malaria;
Research and development of devices for rapid testing of G6PD deficiency and usability evaluation in the field;
Investigation of genetic variants associated with G6PD deficiency in special populations.
**4. Health education and engagement of health workers**
Expansion and strengthening of platforms for training of health professionals and to support the management of malaria cases (TELEMAL+);
Development of qualitative assays to evaluate barriers and facilitators in the use of elimination tools;
Understanding healthcare professionals’ perceptions regarding the usability of elimination tools;
Seeking strategies to improve public engagement in achieving elimination goals.
**5. Strengthening the notification system**
Modernization of the notification system for case registration (SIVEP-Malaria);
Incorporation of machine-learning tools and use of artificial intelligence to study the epidemiological variables available in the notification system;
Integration of the SIVEP-Malaria system with other febrile disease notification systems used in the Brazilian Amazon;
Development of a strategy for the rapid detection and management of reintroduced cases in malaria-free areas.

**ACT:** Artemisinin-based Combination Therapy; **G6PD:** glucose-6-phosphate dehydrogenase; **SIVEP-Malaria:** *Sistema de Informação de Vigilância Epidemiológica de Malária.*

## CONCLUSIONS

Amazonas State has demonstrated a consistent decline in malaria cases and related mortality, despite significant geographical and economic challenges. Since 2003, there has been a 70% reduction in reported malaria cases and a 56% decrease in malaria attributable to *P. falciparum*, reflecting the effectiveness of malaria control and elimination initiatives. Key factors contributing to the elimination goal by 2035 include expanding diagnostic access in remote regions, ensuring safe and effective treatment for all age groups, and implementing coordinated actions among diverse stakeholders and institutions alongside technological advancements. These strategies remain critical for sustaining progress in the fight against malaria.

## Data Availability

Research data is not available.

## References

[1] Sampaio VS, Siqueira AM, Alecrim M das GC, Mourão MPG, Marchesini PB, Albuquerque BC (2015). Malaria in the state of Amazonas: A typical Brazilian tropical disease influenced by waves of economic development. Rev Soc Bras Med Trop.

[2] Ministério da Saúde (MS). Secretaria de Vigilância em Saúde (2025). SIVEP - Malária. Sistema de Informação de Vigilância Epidemiológica - Notificação de Casos.

[3] Melo GC, Monteiro WM, Siqueira AM, Silva SR, Magalhães BML, Alencar ACC (2014). Expression levels of pvcrt-o and pvmdr-1 are associated with chloroquine resistance and severe Plasmodium vivax malaria in patients of the Brazilian Amazon. PLoS One.

[4] Llanos-Cuentas A, Lacerda MVG, Hien TT, Vélez ID, Namaik-larp C, Chu CS (2019). Tafenoquine versus Primaquine to Prevent Relapse of Plasmodium vivax Malaria. N Engl J Med.

[5] Lacerda MVG, Llanos-Cuentas A, Krudsood S, Lon C, Saunders DL, Mohammed R (2019). Single-Dose Tafenoquine to Prevent Relapse of Plasmodium vivax Malaria. N Engl J Med.

[6] Fabbri C, Trindade AO, Andrade FS, de Souza MF, Ríos-Velásquez CM, de Lacerda MVG (2021). Transmission-blocking compound candidates against Plasmodium vivax using P. berghei as an initial screening. Mem Inst Oswaldo Cruz.

[7] Brito MAM, Baro B, Raiol TC, Ayllon-Hermida A, Safe IP, Deroost K (2022). Morphological and Transcriptional Changes in Human Bone Marrow during Natural Plasmodium vivax Malaria Infections. J Infect Dis.

[8] Yakubu MN, Mwangi VI, Netto RLA, Alecrim MGC, Alves JRS, Almeida ACG (2024). Host metabolomic responses in recurrent P. vivax malaria. Sci Rep.

[9] World Health Organization (2025). World malaria report 2025: addressing the threat of antimalarial drug resistance.

[10] Ministério da Saúde (MS). Secretaria de Vigilância em Saúde e Ambiente (2024). Boletim epidemiológico Nº 14: Caracterização da malária em áreas especiais da região amazônica.

[11] Ministério da Saúde (MS). Secretaria de Vigilância em Saúde (2022). Elimina Malária Brasil: Plano Nacional de Eliminação da Malária.

[12] Fundação de Vigilância em Saúde - Dra. Rosemary Costa Pinto (FVS-RCP) (2023). Plano Estadual de Eliminação da Malária no Amazonas 2023-2035.

[13] Machado MB, Figueira EA, Passos RA, Fernandes C, Albuquerque BC, Mutis MCS, Treptow TC (2022). Promoção da saúde e qualidade de vida.

[14] Baia-da-Silva DC, Brito-Sousa JD, Rodovalho SR, Peterka C, Moresco G, Mesones Lapouble OM (2019). Current vector control challenges in the fight against malaria in Brazil. Rev Soc Bras Med Trop.

[15] Ministério da Saúde (MS). Secretaria de Vigilância em Saúde (2021). Guia de tratamento da malária no Brasil.

[16] Brito M, Rufatto R, Murta F, Sampaio V, Balieiro P, Baía-Silva D (2024). Operational feasibility of Plasmodium vivax radical cure with tafenoquine or primaquine following point-of-care, quantitative glucose-6-phosphate dehydrogenase testing in the Brazilian Amazon: a real-life retrospective analysis. Lancet Glob Health.

[17] Ministério da Saúde (MS). Secretaria de Vigilância em Saúde e Ambiente (2024). Boletim epidemiológico Nº 12: Implementação da tafenoquina nos municípios prioritários do Brasil, 2024.

[18] Palma-Cuero M, Machado MB, Graça JTB, Anjos NBD, Pereira RS, Suárez-Mutis MC (2022). Malaria at international borders: challenges for elimination on the remote Brazil-Peru border. Rev Inst Med Trop Sao Paulo.

[19] Albuquerque HG, Santos GBG, Siqueira ASP, Coelho RR, Dos Santos JPC, Praça HLF (2025). Mapping special areas of the Brazilian National Malaria Control Program in the Amazon region: a territorial-based approach to surveillance. Mem Inst Oswaldo Cruz.

[20] Ferreira MU, Castro MC (2016). Challenges for malaria elimination in Brazil. Malar J.

[21] Martins-Campos KM, Kuehn A, Almeida A, Duarte APM, Sampaio VS, Rodriguez ÍC (2018). Infection of Anopheles aquasalis from symptomatic and asymptomatic Plasmodium vivax infections in Manaus, western Brazilian Amazon. Parasit Vectors.

[22] Alencar RM, Sepulveda CCP, Martinez-Villegas L, Bahia AC, Santana RA, de Souza IB (2023). Unravelling the genome of the brackish water malaria vector Anopheles aquasalis. Sci Rep.

[23] Santana RAG, Oliveira MC, Cabral I, Junior RCAS, De Sousa DRT, Ferreira L (2019). Anopheles aquasalis transcriptome reveals autophagic responses to Plasmodium vivax midgut invasion. Parasit Vectors.

[24] Fabbri C, Quaresma Ramos G, Clarys Baia-da-Silva D, Oliveira Trindade A, Carlos Salazar-Alvarez L, Costa Ferreira Neves J (2023). The activity of methylene blue against asexual and sexual stages of Plasmodium vivax. Front Cell Infect Microbiol.

[25] Yamamoto Y, Fabbri C, Okuhara D, Takagi R, Kawabata Y, Katayama T (2024). A two-dose viral-vectored Plasmodium vivax multistage vaccine confers durable protection and transmission-blockade in a pre-clinical study. Front Immunol.

[26] Yamamoto Y, Katayama T, Fabbri C, Niwa S, Okuhara D, Iyori M (2025). Malaria bivalent viral vectored vaccine protects against Plasmodium falciparum and vivax and blocks parasite transmission. NPJ Vaccines.

[27] Martinez EG, Alencar RM, Santana RAG, Barbosa LRA, Almeida ACG, Mwangi VI (2025). Relative efficacy of anti-Plasmodium vivax malaria combination drugs in preventing transmission to two major Anopheles mosquitoes in the first few days of treatment. Int J Infect Dis.

[28] Alexandre MA, Ferreira CO, Siqueira AM, Magalhães BL, Mourão MP, Lacerda MV (2010). Severe Plasmodium vivax malaria, Brazilian Amazon. Emerg Infect Dis.

[29] Siqueira AM, Lacerda MVG, Magalhães BML, Mourão MPG, Melo GC, Alexandre MAA (2015). Characterization of Plasmodium vivax-associated admissions to reference hospitals in Brazil and India. BMC Med.

[30] Park SY, Park YS, Park Y, Kwak YG, Song JE, Lee KS (2019). Severe vivax malaria in the Republic of Korea during the period 2000 to 2016. Travel Med Infect Dis.

[31] Kojom Foko LP, Arya A, Sharma A, Singh V (2021). Epidemiology and clinical outcomes of severe Plasmodium vivax malaria in India. J Infect.

[32] Chu CS, Stolbrink M, Stolady D, Saito M, Beau C, Choun K (2023). Severe Falciparum and Vivax Malaria on the Thailand-Myanmar Border: A Review of 1503 Cases. Clin Infect Dis.

[33] Mendonça VRR, Souza LCL, Garcia GC, Magalhães BML, Gonçalves MS, Lacerda MVG (2015). Associations between hepcidin and immune response in individuals with hyperbilirubinaemia and severe malaria due to Plasmodium vivax infection. Malar J.

[34] Val F, Avalos S, Gomes AA, Zerpa JEA, Fontecha G, Siqueira AMH (2017). Are respiratory complications of Plasmodium vivax malaria an underestimated problem?. Malar J.

[35] Elizalde-Torrent A, Val F, Azevedo ICC, Monteiro WM, Ferreira LCL, Fernández-Becerra C (2018). Sudden spleen rupture in a Plasmodium vivax-infected patient undergoing malaria treatment. Malar J.

[36] Dos-Santos JCK, Silva-Filho JL, Judice CC, Kayano ACAV, Aliberti J, Khouri R (2020). Platelet disturbances correlate with endothelial cell activation in uncomplicated Plasmodium vivax malaria. PLoS Negl Trop Dis.

[37] Costa AG, Chaves YO, Teixeira-Carvalho A, Ramasawmy R, Antonelli LRV, Barbosa L (2020). Increased platelet distribution width and reduced il-2 and il-12 are associated with thrombocytopenia in Plasmodium vivax malaria. Mem Inst Oswaldo Cruz.

[38] Santos RO dos, Cruz MGS da, Lopes SCP, Oliveira LB, Nogueira PA, Lima ES (2020). A First Plasmodium vivax Natural Infection Induces Increased Activity of the Interferon Gamma-Driven Tryptophan Catabolism Pathway. Front Microbiol.

[39] Chaves YO, Da Costa AG, Pereira MLM, De Lacerda MVG, Coelho-Dos-Reis JG, Martins-Filho OA (2016). Immune response pattern in recurrent Plasmodium vivax malaria. Malar J.

[40] Shuan Laco AC, Chaves YO, de Almeida ACG, Farias ES, Mwangi VI, Vallejos MVG (2025). IFN-γ Production in Memory CD4 + T Cells in Response to MSP119 Antigen and Its Correlation with Anemia and Thrombocytopenia in Pediatric Vivax Malaria. ACS Omega.

[41] Benzecry SG, Alexandre MA, Vítor-Silva S, Salinas JL, De Melo GC, Marinho HA (2016). Micronutrient deficiencies and Plasmodium vivax malaria among children in the Brazilian Amazon. PLoS One.

[42] Ferreira EDA, Alexandre MA, Salinas JL, De Siqueira AM, Benzecry SG, De Lacerda MVG (2015). Association between anthropometry-based nutritional status and malaria: A systematic review of observational studies. Malar J.

[43] Monteiro WM, Alexandre MA, Siqueira A, Melo G, Romero GAS, D’Ávila E (2016). Could Plasmodium vivax malaria trigger malnutrition? Revisiting the bradford hill criteria to assess a causal relationship between two neglected problems. Rev Soc Bras Med Trop.

[44] Tapajós R, Castro D, Melo G, Balogun S, James M, Pessoa R (2019). Malaria impact on cognitive function of children in a peri-urban community in the Brazilian Amazon. Malar J.

[45] Pessoa RC, Oliveira-Pessoa GF, Souza BKA, Sampaio VS, Pinto ALCB, Barboza LL (2022). Impact of Plasmodium vivax malaria on executive and cognitive functions in elderlies in the Brazilian Amazon. Sci Rep.

[46] Bardají A, Martínez-Espinosa FE, Arévalo-Herrera M, Padilla N, Kochar S, Ome-Kaius M (2017). Burden and impact of Plasmodium vivax in pregnancy: A multi-centre prospective observational study. PLoS Negl Trop Dis.

[47] Bôtto-Menezes C, Dos Santos MCS, Simplício JL, De Medeiros JM, Gomes KCB, De Carvalho Costa IC (2015). Plasmodium vivax malaria in pregnant women in the Brazilian Amazon and the risk factors associated with prematurity and low birth weight: A descriptive study. PLoS One.

[48] Requena P, Rui E, Padilla N, Martínez-Espinosa FE, Castellanos ME, Bôtto-Menezes C (2016). Plasmodium vivax VIR Proteins Are Targets of Naturally-Acquired Antibody and T Cell Immune Responses to Malaria in Pregnant Women. PLoS Negl Trop Dis.

[49] Requena P, Arévalo-Herrera M, Menegon M, Martínez-Espinosa FE, Padilla N, Bôtto-Menezes C (2017). Naturally acquired binding-inhibitory antibodies to Plasmodium vivax duffy binding protein in pregnant women are associated with higher birth weight in a multicenter study. Front Immunol.

[50] Dobaño C, Bardají A, Arévalo-Herrera M, Martínez-Espinosa FE, Bôtto-Menezes C, Padilla N (2020). Cytokine signatures of Plasmodium vivax infection during pregnancy and delivery outcomes. PLoS Negl Trop Dis.

[51] Dobaño C, Bardají A, Kochar S, Kochar SK, Padilla N, López M (2020). Blood cytokine, chemokine and growth factor profiling in a cohort of pregnant women from tropical countries. Cytokine.

[52] Brock MF, Miranda AE, Bôtto-Menezes C, Leão JRT, Martinez-Espinosa FE (2015). Ultrasound findings in pregnant women with uncomplicated vivax malaria in the Brazilian Amazon: A cohort study. Malar J.

[53] Machado Filho AC, Da Costa EP, Da Costa EP, Reis IS, Fernandes EAC, Paim BV (2014). Effects of vivax malaria acquired before 20 weeks of pregnancy on subsequent changes in fetal growth. Am J Trop Med Hyg.

[54] Bôtto-Menezes C, Bardají A, dos Santos Campos G, Fernandes S, Hanson K, Martínez-Espinosa FE (2016). Costs Associated with Malaria in Pregnancy in the Brazilian Amazon, a Low Endemic Area Where Plasmodium vivax Predominates. PLoS Negl Trop Dis.

[55] Salinas JL, Kissinger JC, Jones DP, Galinski MR (2014). Metabolomics in the fight against malaria. Mem Inst Oswaldo Cruz.

[56] Yu X, Feng G, Zhang Q, Cao J (2021). From Metabolite to Metabolome: Metabolomics Applications in Plasmodium Research. Front Microbiol.

[57] Uppal K, Salinas JL, Monteiro WM, Val F, Cordy RJ, Liu K (2017). Plasma metabolomics reveals membrane lipids, aspartate/asparagine and nucleotide metabolism pathway differences associated with chloroquine resistance in Plasmodium vivax malaria. PLoS One.

[58] Yakubu MN, Mwangi VI, Almeida ACG, Lira E, Adrião AAX, Santos GFD (2025). Metabolic disruptions in P. vivax malaria: Insights from four antimalarial treatment regimens. Acta Trop.

[59] Baro B, Deroost K, Raiol T, Brito M, Almeida ACG, de Menezes-Neto A (2017). Plasmodium vivax gametocytes in the bone marrow of an acute malaria patient and changes in the erythroid miRNA profile. PLoS Negl Trop Dis.

[60] Monteiro WM, Val FFA, Siqueira AM, Franca GP, Sampaio VS, Melo GC (2014). G6PD deficiency in Latin America: Systematic review on prevalence and variants. Mem Inst Oswaldo Cruz.

[61] Nascimento JR, Brito-Sousa JD, Almeida ACG, Melo MM, Costa MRF, Barbosa LRA (2022). Prevalence of glucose 6-phosphate dehydrogenase deficiency in highly malaria-endemic municipalities in the Brazilian Amazon: A region-wide screening study. Lancet Reg Health Am.

[62] Brito-Sousa JD, Santos TC, Avalos S, Fontecha G, Melo GC, Val F (2019). Clinical Spectrum of Primaquine-induced Hemolysis in Glucose-6-Phosphate Dehydrogenase Deficiency: A 9-Year Hospitalization-based Study from the Brazilian Amazon. Clin Infect Dis.

[63] Brito-Sousa JD, Peixoto HM, Devine A, Silva-Neto AV, Balieiro PCS, Sampaio VS (2022). Real-life quantitative G6PD screening in Plasmodium vivax patients in the Brazilian Amazon: A cost-effectiveness analysis. PLoS Negl Trop Dis.

[64] Brito-Sousa JD, Murta F, Vitor-Silva S, Sampaio VS, Mendes MO, Brito MAM (2021). Real-life implementation of a g6pd deficiency screening qualitative test into routine vivax malaria diagnostic units in the Brazilian amazon (Safeprim study). PLoS Negl Trop Dis.

[65] Brito-Sousa JD, Phanor J, Balieiro PCDS, Silva-Neto AV, Cordeiro JSM, Vitor-Silva S (2022). Effect of weekly versus daily primaquine on Plasmodium vivax malaria recurrences: A real-life cohort study. Rev Soc Bras Med Trop.

[66] Barbosa L, Brito-Sousa J, Nascimento C, Pacheco A, Alexandre M, Alencar A (2025). Safety and efficacy of three alternative regimens against relapsing Plasmodium vivax malaria in glucose-6-phosphate dehydrogenase deficient patients in the Brazilian Amazon (ALTPRIM). Clin Infect Dis.

[67] Mehdipour P, Rajasekhar M, Dini S, Zaloumis S, Abreha T, Adam I (2023). Effect of adherence to primaquine on the risk of Plasmodium vivax recurrence: a WorldWide Antimalarial Resistance Network systematic review and individual patient data meta-analysis. Malar J.

[68] Daher A, Silva JCAL, Stevens A, Marchesini P, Fontes CJ, Ter Kuile FO (2019). Evaluation of Plasmodium vivax malaria recurrence in Brazil. Malar J.

[69] Dinelly KMO, Vitor-Silva S, Brito-Sousa JD, Sampaio VS, Silva MGO, Siqueira AM (2021). Evaluation of the effect of supervised anti-malarial treatment on recurrences of Plasmodium vivax malaria. Malar J.

[70] Santos APC, Brito MAM, Oliveira APS, Dávila RN, Gama HSS, Silva EART (2024). Assessing tafenoquine implementation in Brazil: a qualitative evaluation of perceptions of healthcare providers and Plasmodium vivax patients (QualiTRuST Study). Malar J.

[71] Santos A, Brito M, Silva E, Rocha F, Oliveira A, Dávila R (2024). Perspectives of healthcare professionals on training for quantitative G6PD testing during implementation of tafenoquine in Brazil (QualiTRuST Study). PLoS Negl Trop Dis.

[72] Brito M, Rufatto R, Brito-Sousa JD, Murta F, Sampaio V, Balieiro P (2024). Operational effectiveness of tafenoquine and primaquine for the prevention of Plasmodium vivax recurrence in Brazil: a retrospective observational study. Lancet Infect Dis.

[73] Almeida ACG, Kuehn A, Castro AJM, Vitor-Silva S, Figueiredo EFG, Brasil LW (2018). High proportions of asymptomatic and submicroscopic Plasmodium vivax infections in a peri-urban area of low transmission in the Brazilian Amazon. Parasit Vectors.

[74] Barbosa LRA, da Silva EL, de Almeida ACG, Salazar YEAR, Siqueira AM, Alecrim MDGC (2020). An ultra-sensitive technique: Using pv-mtcox1 qPcr to detect early recurrences of Plasmodium vivax in patients in the Brazilian amazon. Pathogens.

[75] Das D, Vongpromek R, Assawariyathipat T, Srinamon K, Kennon K, Stepniewska K (2022). Field evaluation of the diagnostic performance of EasyScan GO: a digital malaria microscopy device based on machine-learning. Malar J.

[76] World Health Organization (2016). Malaria Microscopy Quality Assurance Manual.

[77] de Melo GC, Netto RLA, Mwangi VI, Salazar YEAR, de Souza Sampaio V, Monteiro WM (2021). Performance of a sensitive haemozoin-based malaria diagnostic test validated for vivax malaria diagnosis in Brazilian Amazon. Malar J.

[78] Ministério da Saúde (MS). Secretaria de Vigilância em Saúde (2025). TeleMal.

[79] Zanger UM, Schwab M (2013). Cytochrome P450 enzymes in drug metabolism: Regulation of gene expression, enzyme activities, and impact of genetic variation. Pharmacol Ther.

[80] Sortica VA, Lindenau JD, Cunha MG, Ohnishi MDO, Ventura AMR, Ribeiro-Dos-Santos AK (2016). The effect of SNPs in CYP450 in chloroquine/primaquine Plasmodium vivax malaria treatment. Pharmacogenomics.

[81] Silvino ACR, Costa GL, Araujo FCF de, Ascher DB, Pires DEV, Fontes CJF (2016). Variation in Human Cytochrome P-450 Drug-Metabolism Genes: A Gateway to the Understanding of Plasmodium vivax Relapses. PLoS One.

[82] Brasil LW, Rodrigues-Soares F, Santoro AB, Almeida ACG, Kühn A, Ramasawmy R (2018). CYP2D6 activity and the risk of recurrence of Plasmodium vivax malaria in the Brazilian Amazon: a prospective cohort study. Malar J.

[83] Silvino ACR, Kano FS, Costa MA, Fontes CJF, Soares IS, de Brito CFA (2020). Novel Insights into Plasmodium vivax Therapeutic Failure: CYP2D6 Activity and Time of Exposure to Malaria Modulate the Risk of Recurrence. Antimicrob Agents Chemother.

[84] Almeida ACG, Puça MCB, Figueiredo EFG, Barbosa LR, Salazar YEAR, Silva EL (2020). Influence of CYP2C8, CYP3A4 and CYP3A5 host genotypes on early recurrence of Plasmodium vivax. Antimicrob Agents Chemother.

[85] Almeida AC, Elias ABR, Marques MP, de Melo GC, da Costa AG, Figueiredo EFG (2021). Impact of Plasmodium vivax malaria and antimalarial treatment on cytochrome P450 activity in Brazilian patients. Br J Clin Pharmacol.

[86] Cardoso JLM, Salazar YEAR, Almeida ACG, Barbosa LRA, Silva EL, Rodrigues MGA (2022). Influence of CYP2D6, CYP3A4 and CYP2C19 Genotypes on Recurrence of Plasmodium vivax. Front Trop Dis.

[87] Macêdo MM, Almeida ACG, Silva GS, Oliveira AC, Mwangi VI, Shuan AC (2023). Association of CYP2C19, CYP2D6 and CYP3A4 Genetic Variants on Primaquine Hemolysis in G6PD-Deficient Patients. Pathogens.

[88] Puça MCSB, Rodrigues DF, Salazar YEAR, Louzada J, Fontes CJF, Daher A (2024). Monoamine oxidase-A (MAO-A) low-expression variants and increased risk of Plasmodium vivax malaria relapses. J Antimicrob Chemother.

[89] da Silva GS, Fontenelle FA, Carvalho AO, Macêdo MM, Morais MC, Abreu Netto RL (2025). Impact of CYP2D6, MAOA, and UGT2B7 genetic variants on recurrence of Plasmodium vivax in the Brazilian Amazon. Sci Rep.

[90] St Jean PL, Xue Z, Carter N, Koh GCKW, Duparc S, Taylor M (2016). Tafenoquine treatment of Plasmodium vivax malaria: Suggestive evidence that CYP2D6 reduced metabolism is not associated with relapse in the Phase 2b DETECTIVE trial. Malar J.

[91] Suarez-Kurtz G (2021). Impact of CYP2D6 Genetic Variation on Radical Cure of Plasmodium vivax Malaria. Clin Pharmacol Ther.

[92] Chehuan YF, Costa MR, Costa JS, Alecrim MG, Nogueira F, Silveira H (2013). In vitro chloroquine resistance for Plasmodium vivax isolates from the Western Brazilian Amazon. Malar J.

[93] Marques MM, Costa MRF, Santana Filho FS, Vieira JLF, Nascimento MTS, Brasil LW (2014). Plasmodium vivax chloroquine resistance and anemia in the western Brazilian amazon. Antimicrob Agents Chemother.

[94] Price RN, von Seidlein L, Valecha N, Nosten F, Baird JK, White NJ (2014). Global extent of chloroquine-resistant Plasmodium vivax: a systematic review and meta-analysis. Lancet Infect Dis.

[95] Silva SR, Almeida ACG, Da Silva GAV, Ramasawmy R, Lopes SCP, Siqueira AM (2018). Chloroquine resistance is associated to multi-copy pvcrt-o gene in Plasmodium vivax malaria in the Brazilian Amazon. Malar J.

[96] Siqueira AM, Alencar AC, Melo GC, Magalhaes BL, Machado K, Filho ACA (2017). Fixed-dose artesunate-amodiaquine combination vs chloroquine for treatment of uncomplicated blood stage P. vivax infection in the Brazilian amazon: An open-label randomized, controlled trial. Clin Infect Dis.

[97] Lucchi NW, Abdallah R, Louzada J, Udhayakumar V, Oliveira-Ferreira J (2020). Molecular surveillance for polymorphisms associated with artemisinin-based combination therapy resistance in Plasmodium falciparum isolates collected in the State of Roraima, Brazil. Am J Trop Med Hyg.

[98] Mathieu LC, Singh P, Monteiro WM, Magris M, Cox H, Lazrek Y (2021). Kelch13 mutations in Plasmodium falciparum and risk of spreading in Amazon basin countries. J Antimicrob Chemother.

[99] Alho RM, Machado KVA, Val FFA, Fraiji NA, Alexandre MAA, Melo GC (2017). Alternative transmission routes in the malaria elimination era: an overview of transfusion-transmitted malaria in the Americas. Malar J.

[100] Recht J, Siqueira AM, Monteiro WM, Herrera SM, Herrera S, Lacerda MVG (2017). Malaria in Brazil, Colombia, Peru and Venezuela: Current challenges in malaria control and elimination. Malar J.

[101] Prusty D, Gupta N, Upadhyay A, Dar A, Naik B, Kumar N (2021). Asymptomatic malaria infection prevailing risks for human health and malaria elimination. Infect Genet Evol.

[102] Ministério da Saúde (MS). Coordenação-Geral de Sangue e Hemoderivados (2023). NOTA TÉCNICA No 49/2023-CGSH/DAET/SAES/MS: Processo de implantação do teste para detecção de ácido nucléico de HIV /HBV/HCV/Malária (kit NAT PLUS) e renovação do parque tecnológico nos 14 Sítios Testadores da Hemorrede Nacional.

[103] Rocha D, De Melo GC, Carneiro JMH, Ribeiro M, Ribeiro S, De Godoy DT (2020). Use of a NAT-based assay to improve the surveillance system and prevent transfusion-transmitted malaria in blood banks. Malar J.

[104] Fundação Oswaldo Cruz (FIOCRUZ) (2022). Instituto de Tecnologia em Imunobiológicos (Biomanguinhos/FIOCRUZ). Kit NAT PLUS HIV/HBV/HCV/Malária: instruções de uso.

[105] Costa E, Rocha D, Lopes JIF, Andrade E, Cardoso P, Ribeiro M (2024). Detection of Plasmodium spp. in asymptomatic blood donors by the new Brazilian NAT PLUS HIV/HBV/HCV/Malaria Bio-Manguinhos kit. Transfusion.

[106] Pinheiro TCP, Santos SS, Simião FMEB, Mello ARL, Pimentel CB, Lomonaco LA (2024). Molecular test for screening malaria-infected blood donors to maximise recipient safety in Acre State, a Brazilian endemic area. Mem Inst Oswaldo Cruz.

[107] Gomes de Almeida AC, Shuan Laco AC, Rodrigues MG, Godinho de Siqueira VG, de Moura Brito FB, Gato CM (2026). Improving blood safety: NAT-based detection of Plasmodium spp. in blood donors in endemic areas of Brazil. Blood Transfus.

